# Early risk factors for conduct problem trajectories from childhood to adolescence: the 2004 Pelotas (BRAZIL) Birth Cohort

**DOI:** 10.1007/s00787-023-02178-9

**Published:** 2023-04-25

**Authors:** Thais Martins-Silva, Andreas Bauer, Alicia Matijasevich, Tiago N. Munhoz, Aluísio J. D. Barros, Iná S. Santos, Luciana Tovo-Rodrigues, Joseph Murray

**Affiliations:** 1 Human Development and Violence Research Centre (DOVE), Federal University of Pelotas, Pelotas, Brazil; 2 Post-Graduate Program in Epidemiology, Federal University of Pelotas, Pelotas, Brazil; 3 Departamento de Medicina Preventiva, Faculdade de Medicina FMUSP, Universidade de São Paulo, São Paulo, Brazil; 4 International Center for Equity in Health, Federal University of Pelotas, Pelotas, Brazil; 5 Postgraduate Program in Pediatrics and Child Health, School of Medicine, Pontifical Catholic University of Rio Grande Do Sul, Porto Alegre, Brazil

**Keywords:** Conduct problems, Antisocial behavior, Developmental trajectories, Early risk factors, Birth Cohort, Brazil

## Abstract

Conduct problems are associated with an increased risk of a wide range of physical, mental, and social problems. However, there is still uncertainty about how early risk factors differentiate different developmental patterns of conduct problems and whether findings replicate across diverse social contexts. We aimed to identify developmental trajectories of conduct problems, and test early risk factors, in the 2004 Pelotas Birth Cohort in Brazil. Conduct problems were measured at ages 4, 6, 11, and 15 years from caregiver reports on the Child Behaviour Checklist (CBCL) and Strengths and Difficulties Questionnaire (SDQ). Conduct problem trajectories were estimated using group-based semi-parametric modeling (*n* = 3938). Multinomial logistic regression was used to examine associations between early risk factors and conduct problem trajectories. We identified four trajectories: three with elevated conduct problems, including early-onset persistent (*n* = 150; 3.8%), adolescence-onset (*n* = 286; 17.3%), and childhood-limited (*n* = 697; 17.7%), and one with low conduct problems (*n* = 2805; 71.2%). The three elevated conduct problem trajectories were associated with a wide range of sociodemographic risk factors, prenatal smoking, maternal mental health, harsh parenting, childhood trauma, and child neurodevelopmental risk factors. Early-onset persistent conduct problems were particularly associated with trauma, living without a father figure, and attention difficulties. The four trajectories of conduct problems from ages 4 to 15 years in this Brazilian cohort have similar longitudinal patterns to those identified in high-income countries. The results confirm previous longitudinal research and developmental taxonomic theories on the etiology of conduct problems in a Brazilian sample.

**Supplementary Information:**

The online version contains supplementary material available at 10.1007/s00787-023-02178-9.

## Introduction

Conduct problems refer to aggressive and antisocial behaviors, such as lying, fighting, truancy, and stealing, in childhood and/or adolescence, that are symptomatic of oppositional defiant disorder and conduct disorder [1]. Frequent and persistent conduct problems are associated with an increased risk of a wide range of physical, mental, and social problems throughout the life-course, resulting in pervasive costs for individuals, families, and societies [2–6]. Given this prognosis, identifying modifiable early risk factors that could be targeted in preventive interventions has been an important area of research. Although considerable evidence has accumulated in high-income countries (HICs) on early risk factors, little research has been conducted in low- and middle-income countries (LMICs), where most of the world’s children reside with large variations in sociocultural contexts.

According to Moffitt’s influential developmental taxonomic theory [7], as originally proposed, conduct problems can be categorized into two main groups, with fundamentally different patterns of development across the life-course, and distinct origins. In this theory, *early-onset persistent* (also called ‘life-course persistent’) conduct problems are proposed to emerge in childhood and persist through adolescence into adulthood. These childhood-onset conduct problems are primarily driven by accumulating negative interactions between children with high-risk characteristics (i.e., inherited or acquired neuropsychological deficits) and high-risk social environments. In a cumulating cascade of difficult behavior, children on this pathway experience strained relationships, first with caregivers, and subsequently at school with their teachers and peers, before engaging in more serious antisocial behavior during late adolescence and in adulthood. By contrast, *adolescence-limited* conduct problems are proposed to emerge first in adolescence, mainly as a result of delinquent peer relationships and the discrepancy between adolescents’ biological maturity and their limited access to the privileges and responsibilities of adults. According to Moffitt’s theory, these behaviors naturally decline with new social roles in young adulthood. Thus, while early-onset persistent conduct problems are considered pervasive and pathological, adolescence-limited conduct problems are viewed as transient and normative. Other theoretical models suggest that conduct problems have other key distinguishing characteristics, such as whether or not callous-unemotional traits (or “limited prosocial emotions”) are present, which indicate more serious and persistent behavior problems, including premeditated aggression, and poorer treatment outcomes [8, 9].

Although longitudinal research has documented important bio-psycho-social risk factors for conduct problems in general [10], identifying risk factors for specific conduct problem trajectories has been more challenging, and nearly all research has been limited to HICs [11, 12]. The Dunedin (New Zealand) longitudinal study was the first to demarcate important early differences between early-onset persistent and adolescent-onset groups, particularly in terms of childhood neuropsychological functioning and adverse family environments [13]. In a meta-analysis of further studies, Assink et al. [14] identified elevated psychosocial risk factors including aggression, alcohol/drug abuse, sexual, emotional, and behavioral problems distinguishing early-onset persistent and adolescence-limited conduct problems, although family environment had only weak effects. Notably, in this meta-analysis, out of 55 included studies, 54 were conducted in HICs, mainly in the US. Similarly, in another systematic review comparing early risk factors for long-term trajectories of early-onset persistent, adolescence-limited, and late-onset offenders, only longitudinal studies from HICs (the US and UK) were included [15]. The Dunedin study was the only one in that review including data from early childhood, and other studies that had started from age 8 years reported relatively weak differences in risk factors across trajectory groups. Thus, questions remain about the importance of early environment and child characteristics for distinguishing different trajectories of antisocial behavior, and there is a very significant gap in evidence in LMICs, with different cultures, levels of economic deprivation, and in some regions, very high rates of community violence, compared to HICs.

The lack of evidence from the Global South is further illustrated in a systematic review specifically focusing on risk factors for antisocial behavior in LMICs—extensive searches in seven languages located just one study that examined conduct problem trajectories [16]. This study compared conduct problem trajectories across ages 10–12 years among children from San Juan (Puerto Rico) and New York City [17]. While this study provides initial evidence that risk factors may be more similar than different across cultural contexts, it did not include data from early life, but defined trajectories across only three years of early adolescence. Ideally, new studies in LMICs would explore the full range of possible risk factors, including in early life, for different conduct problem trajectories mapped across the life-course. Specifically, considering the sociodemographic domain, it would be desirable to consider whether factors such as family poverty, parental education and age, and father presence associate with persistent conduct problems, as has been found in studies in HICs, such as the Dunedin longitudinal study [18]. Prenatal risk factors such as smoking in pregnancy should be tested, as well as quality of parenting practices, and maternal depression, which have been implicated in developmental models of conduct problems in HICs [18]. Considering the extensive literature relating maltreatment and other forms of victimization to antisocial behaviors [19, 20], interpersonal trauma is another important potential risk for the development and persistence of conduct problems. Finally, considering individual characteristics of the child, tests of neuropsychological functioning (e.g., IQ) should be tested, as theorized by Moffitt as critical in early-onset persistent conduct problems [18], as well as the role of attention-deficit hyperactivity symptoms, which commonly co-occur with conduct problems [21, 22], as well as callous-unemotional traits.

Brazil, where this study was conducted, is a middle-income country characterized by high rates of violence and deep social inequalities, with homicide rates some 25 times higher than in many HICs. Interpersonal violence is the leading cause of death among young people in Brazil [23, 24]. Longitudinal studies in Brazil have already demonstrated that high levels of child conduct problems are associated with later interpersonal violence [25], however, no data are available on developmental patterns of conduct problems in Brazil or their risk factors. We studied developmental trajectories of conduct problems from ages 4 to 15 years in a Brazilian birth cohort to determine whether similar conduct problem trajectories would be identified as elsewhere and whether early risk factors across different domains (including sociodemographic, prenatal, maternal, and child mental health, parenting, childhood trauma, and neurocognitive characteristics) differentiate conduct problem trajectories in this setting. We expected that similar types of conduct problem trajectories would be identified in our Brazilian birth cohort as elsewhere, but considered that trajectories with elevated problems (particularly early-onset persistent and adolescent-onset conduct problems) might be more prevalent in this relatively disadvantaged, and violent setting. Given that previous studies in LMICs have found many similar correlates of conduct problems (without analyzing trajectories) as in HIC studies [26], we hypothesized that risk factors for conduct problem trajectories will also be quite similar to those identified in HIC contexts.

## Methods

### Study design

The 2004 Pelotas (Brazil) Birth Cohort is a population-based, prospective study, including all children born in 2004 in Pelotas, a southern Brazilian city of approximately 340,000 people. All women with live births residing in the urban area of the city (*n* = 4261) were invited to participate. Of those women, 4231 (99.3%) gave informed consent and were included in the study with their children. Trained interviewers collected information on maternal and child health within 24 h after delivery using a structured questionnaire, and all newborns were examined by a pediatrician. Since the perinatal visit, mothers and their children have been assessed again at 3 months and 1, 2, and 4 years, at home, and 6 and 11 years at a research clinic run by the Postgraduate Program in Epidemiology at the Federal University of Pelotas. At the age of 15 years, follow-up assessments were completed in the research clinic as well as via telephone due to the Covid-19 pandemic, which interrupted in-person assessments after about half of the cohort had been interviewed. Mental health outcomes were measured by psychologists and RedCap was used as the instrument for data collection [27]. More details about the cohort methodology have been described elsewhere [28, 29].

### Measures

#### Conduct problems from childhood to adolescence

During the 4-year follow-up, mothers or caregivers were interviewed by trained interviewers using the Child Behaviour Checklist (CBCL) [30], which has been validated for use in Brazilian children [31]. The instrument consists of eight subscales, including withdrawn, somatic complaints, anxiety/depression, social problems, thought problems, attention problems, and aggressive and rule-breaking behavior (previously called delinquent behavior). Items of the aggressive (20 items) and rule-breaking (17 items) behavior subscales, which are rated on a 3-point scale (from *not true*, *somewhat or sometimes true*, and *very true or often true*), were summed up to create a composite measure of conduct problems (ranging from 0 to 52).

At the 6-, 11- and 15-year follow-ups, the Strengths and Difficulties Questionnaire (SDQ) was completed by mothers or caregivers. The SDQ has been validated for use in Brazil [32] and consists of five subscales, with 5 items each, including conduct problems, emotional problems, hyperactivity/inattention, peer relationship problems, and prosocial behavior. Items are scored on a 3-point scale (*not true*, *somewhat true*, and *certainly true*), and in the current study, we used the conduct problems subscale (ranging from 0 to 10).

As we were interested in estimating conduct problem trajectories from ages 4 to 15 years, we standardized the CBCL and SDQ subscales as z-scores at each time point, to improve the comparability across these measures. The CBCL and SDQ conduct problem scales are highly correlated [33].

#### Early risk factors

In the current study, seven groups of early risk factors were examined. Supplementary Table 1 summarizes the timing of assessment, coding, and the valid number of participants for each risk factor.

1. *Sociodemographic risk factors.* Maternal age (coded as < 19 or ≥ 19 years) was assessed at the perinatal interview. At age 4 years, we used maternal schooling (categorized as 0–4, 5–8, or ≥ 9 years), family income (in tertiles), and household crowding—which was defined as the total number of co-residents per household divided by the total number of rooms used for sleeping (coded as ≤ 2 or > 2 individuals per bedroom), and living without a father figure *(“Does the social [biological or adoptive] father live with the child?”*; coded as *yes* or *no*).
2. *Prenatal risk factors:* Maternal smoking (*“Did you smoke during the pregnancy?”*; coded as *yes* or *no*) and maternal alcohol consumption (*“Did you drink alcohol during the pregnancy?”;* coded as *yes* or *no*), were measured during the perinatal interview.
3. *Maternal mental health:* Maternal depression was assessed using the Edinburgh Postnatal Depression Scale (EPDS) at the 4-year follow-up [34]. The EPDS is a self-report questionnaire, asking about depressive symptoms over the past 7 days. The 10 items are rated on a 4-point scale (ranging from 0 to 30). The Portuguese version of the EPDS has been cross-culturally adapted and validated for use in Brazil [35]. An EPDS score of ≥ 13 was defined as probable depression [35].
4. *Parenting risk factors:* At age 6, harsh parenting was measured by asking mothers or caregivers to complete the parent-to-child version of the Conflict Tactics Scale (CTSPC) [36]. The CTSPC is a self-report questionnaire, asking about harsh parenting behaviors in the past 12 months. The CTSPC consists of 22 items across three subscales: non-violent discipline (4 items), psychological aggression (5 items), and physical assault, which includes corporal punishment (5 items), physical maltreatment (4 items), and severe physical maltreatment (4 items; not used in the current study). The Portuguese version of the CTSPC has been cross-culturally adapted and validated for use in Brazil [37]. In the current study, psychological aggression, corporal punishment, and physical maltreatment subscales were summed to create a total score of harsh parenting, which was divided into tertiles (1st as lower and 3rd as higher). To evaluate child cognitive stimulation, we used five items asking whether someone read or told a story to the child; whether the child went to a park or playground; whether the child went to another person’s house; whether the child watched TV; and whether the child had a storybook at home. In the current study, we used a simple score derived by summing the number of positive answers obtained for these five questions (ranging from 0 to 5) and classified them according to a previous study as 0–2, 3, or 4–5 points [38].
5. *Childhood trauma:* we assessed childhood exposure to interpersonal trauma using the post-traumatic stress disorder section of the Development and Well-Being Assessment at the 6-year follow-up, which was adapted and validated for use in Brazil [32]. A trained psychologist asked caregivers whether their child was exposed to an attack or threat, physical abuse, sexual abuse, or rape; witnessed domestic violence; or witnessed or learned about an attack or threat towards a family member or friend during their lifetime. In the current study, interpersonal trauma was coded as *present* or *absent*.
6. *Child neurocognitive risk factors:* at age 4 years, a trained interviewer administered the screening version of the BDI to evaluate child development in five domains: personal–social, adaptive, motor, communication, and cognitive development. The BDI is a screening tool that comprises 96 items with three administration formats: structured questionnaire, observation, and interviews with parents or other sources, such as the child’s teacher [39]. In line with previous work [38, 40], the BDI scores were dichotomized to define a low child development group using the 10th percentile as the cut-off point (i.e., children with low development belonging to the first decile). At age 6 years, IQ was measured using 4 subtests from the Wechsler Intelligence Scale for Children-III (WISC-III): 2 verbal (similarities and arithmetic) and 2 performances (block building and picture completion) [41, 42]. In the current study, we combined all four scores using the norms previously defined in the US, and a total score of < 70 was defined as low IQ.
7. *Child mental health:* Attention problems were examined as a possible risk factor for child conduct problems, using the attention problems subscale from the CBCL [30, 31] applied at age 4 years (continuous scale ranging from 0 to 16).

### Statistical analysis

The statistical analysis proceeded in four steps. First, we described the prevalence rates of all early risk factors for the total sample. We also compared these rates between participants included in the main analyses and those excluded. Second, conduct problem trajectory models from ages 4 to 15 years were estimated using a group-based trajectory modeling (GBTM), a semi-parametric approach [43]. GBTM is a type of longitudinal finite mixture model designed to identify groups of individuals following similar developmental trajectories. This approach assumes that a population is composed of distinct groups, defined by similar changes over time. In this study, we used a polynomial function in a censored normal model to examine how conduct problems change with age for different groups of children. GBTM handles missing data using maximum likelihood estimation under the missing-at-random (MAR) assumption [44]. Applying this in our study, allowed for the inclusion of participants with data available from at least one follow-up (i.e., with data from at least one follow-up at ages 4, 6, 11 or 15 years). To select the optimal trajectory model, we used the Bayesian Information Criterion (BIC), with lower values indicating better model fit [45], and considered the interpretability of each group trajectory, as well as the average posterior probability (APP) of group membership (values above 0.70 indicating that classes are distinct from each other) [43, 46].

In the third stage of analyses, we examined rates of early risk factors for each conduct problem trajectory, and tested for any overall differences across the groups, using chi-square tests and/or ANOVA. Finally, we examined associations between early risk factors and conduct problem trajectories, using adjusted multinomial logistic regression models, with results presented as odds ratios (ORs) with 95% confidence intervals (95% CIs). Two types of outcome comparisons were made in these regression analyses. In the first comparison, the low conduct problem trajectory was used as the reference group. In the second comparison, the early-onset persistent group was compared to each of the childhood-limited and adolescent-onset trajectories.

Risk factors were added to regression models in five hierarchical levels (see Supplementary Table 1): (i) sociodemographic risk factors; (ii) prenatal risk factors; (iii) maternal mental health; (iv) parenting risk factors and childhood trauma; and (v) child neurocognitive risk factors and child mental health. Risk factors with *p* < 0.20 were kept in the model as possible confounders for subsequent levels.

All analyses were performed with Stata software version 16.1 (StataCorp IC, College Station, Tex), and the trajectories models were estimated using the “*traj*” and “*cnorm*” commands, in the same software.

### Ethics statement

The 2004 Pelotas Birth Cohort study obtained ethical approval for each follow-up from the Medical School Ethics Committee of the Federal University of Pelotas. Mothers were fully informed of all follow-up procedures, the general objectives, the voluntary nature of their participation, their right not to participate, their right not to answer specific questions, and their right to the confidentiality of the information provided. At the 11- and 15-year follow-up, adolescents also signed an informed consent form. Cases of severe mental health problems, as identified by the psychologists, were evaluated and, when necessary, were referred to psychiatric or psychological care facilities.

## Results

### Missing data

Of the 4231 children included in the perinatal study, 101 died up to the age of 11 years. In total, 3869 (91.4%), 3799 (89.8%), 3722 (88.0%), and 3565 (84.3%) participants were interviewed at ages 2, 4, 6 and 11 years, respectively. At 15 years, due to the Covid-19 pandemic, we interviewed 50.4% of the cohort (2029 participants). For the current analyses, participants with missing data on conduct problems at all assessment waves were excluded (*n* = 293), resulting in 3938 participants (95.3% of the original cohort) for whom conduct problem trajectories could be estimated (given only one data point is required for trajectory estimation). At ages 4, 6, 11, and 15 years, there were data available on conduct problems for 3750, 3580, 3563, and 1942 participants, respectively. Conduct problem trajectory models were estimated for 1760 participants with all 4 valid data points, 1631 participants with 3 valid data points, 355 participants with 2 valid data points, and 192 participants with 1 valid data point.

### Sample characteristics

Descriptive statistics for the sample of 3938 children included in the conduct problem trajectory analyses are presented in Table 1. There were slightly more boys (51.9%) than girls in the sample. The mean scores of conduct problems at ages 4, 6, 11, and 15 years were 15.48 (± 0.12 [using CBCL; scores ranging from 0 to 52]), and, using the SDQ (scores ranging from 0 to 10), 1.53 (± 0.03), 1.39 (± 0.03), and 1.40 (± 0.04), respectively. At the time of birth of the cohort child, 14.1% of mothers were < 19 years old, and 15.3% had 4 years or less of schooling. The average monthly family income was R$1,456.2 (around USD 795 at the time of data collection; data not shown). About half of the families lived with more than two people per bedroom (53.2%). Almost one-fourth of mothers (23.1%) reported living without a father figure during the child’s upbringing. In total, 27.1% and 3.3% of mothers smoked and/or consumed alcohol during pregnancy, respectively. Almost one-fifth (17.9%) of mothers had high depression scores. Regarding parenting factors, almost half of the children had high stimulation scores (43.3% had a score of 4 or 5). Almost one-third (31.1%) of children experienced harsh parenting and 5.1% had been exposed to interpersonal trauma. About one-third of children (30.0%) had a low IQ score. The mean score for attention problems was 2.62 (± 0.04). There were no differences between those included and those excluded from the analysis (see Supplementary Table 2).

**Table 1:** Description of the included sample

Variables	Included sample	
	*n*	% (95% CI)
*Sociodemographic risk factors*		
*Maternal age (years)*		
< 19	556	14.1 (13.1; 15.3)
≥ 19	3,379	85.9 (84.7; 86.9)
*Maternal schooling (years)*		
0–4	577	15.3 (14.2; 16.5)
5–8	1,398	37.1 (35.6; 38.6)
≥ 9	1,795	47.6 (46.0; 49.2)
*Family income (in tertiles)*		
1st (poorest)	1,338	35.4 (33.8; 36.9)
2nd	1,191	31.5 (30.0; 33.0)
3rd (richest)	1,256	33.2 (31.7; 34.7)
*Household crowding (individuals per bedroom)*		
> 2	2,012	53.2 (51.6; 54.8)
≤ 2	1,772	46.8 (45.2; 48.4)
*Living without a father figure*		
Yes	874	23.1 (21.8; 24.5)
No	2,911	76.9 (75.5; 78.2)
*Prenatal risk factors*		
*Maternal smoking*		
Yes	1,068	27.1 (25.8; 28.5)
No	2,869	72.9 (71.5; 74.2)
*Maternal alcohol consumption*		
Yes	129	3.3 (2.8; 3.9)
No	3,808	96.7 (96.1; 97.2)
*Maternal mental health*		
Maternal depression		
Yes	670	17.9 (16.7; 19.2)
No	3,065	82.1 (81.0; 83.3)
*Parenting risk factors*		
*Child stimulation score*		
0–2 (lower)	1,013	26.8 (25.4; 28.3)
3	1,128	29.9 (28.4; 31.4)
4–5 (higher)	1,634	43.3 (41.7; 44.9)
*Harsh parenting (in tertiles)*		
3rd (worse)	1,076	31.1 (30.0; 32.7)
2nd	1,160	33.5 (32.0; 35.1)
1st (better)	1,225	35.4 (33.8; 37.0)
*Childhood trauma*		
*Interpersonal trauma*		
Present	183	5.1 (4.4; 5.9)
Absent	3,400	94.9 (94.1; 95.6)
*Child neurocognitive risk factors*		
*Low child development*		
Yes	422	11.2 (10.2; 12.2)
No	3,360	88.8 (87.8; 89.8)
Low IQ		
Yes	1,061	30.0 (28.5; 31.6)
No	2,472	70.0 (68.4; 71.4)
*Child mental health*		
Attention problems subscale (mean[sd])	3,749	2.62 (0.04)
*Child conduct problems (mean[sd])*^*a*^		
At 4 years	3,750	15.48 (0.12)
At 6 years	3,580	1.53 (0.03)
At 11 years	3,563	1.39 (0.03)
At 15 years	1,942	1.40 (0.04)

2004 Pelotas (Brazil) Birth Cohort (*N* = 3938)

95% CI 95% confidence interval, *sd* standard deviation. ^a^At age 4 years, the CBCL (ranging from 0 to 52) was used; for all other time points, we used the SDQ conduct problems subscale (ranging from 0 to 10)

### Developmental trajectories of conduct problems

**Fig. 1:**
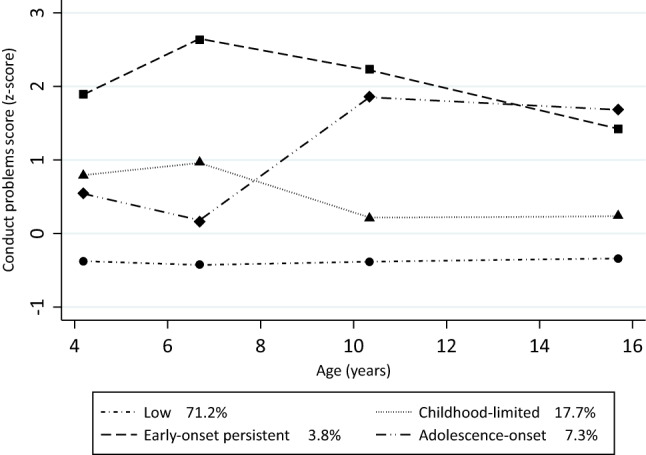
Conduct problem trajectories from ages 4 to 15 years in the 2004 Pelotas (Brazil) Birth Cohort. N = 3,938. Lines represent estimated (latent) change over time. Dots represent observed group means at each age (markers). The square, circle, diamond, and triangle represent the exact z-score of conduct problems in each trajectory at ages 4, 6, 11, and 15 years

We estimated conduct problem trajectories by testing three-, four-, and five-group models. The three-group (BIC -16,782,27) and five-group models (BIC -16,501,72) showed poorer model fit compared to the four-group model (BIC -16,360,50), and added difficulty in interpreting the derived trajectories. Therefore, the four-group model using a cubic pattern of change emerged as the best fitting and most parsimonious model. For all four conduct problem groups, the APP was above the recommended threshold for assignment of 0.70 (APPs ranging from 0.78 to 0.93; see Supplementary Table 3). Figure 1 shows the four-group model: group 1 (labeled “early-onset persistent”, *n* = 150) comprised 3.8% of the children, group 2 (labeled “adolescence-onset”, *n* = 286) comprised 7.3% of the children, group 3 (labeled “childhood-limited”, *n* = 697) comprised 17.7% of the children, and group 4 (labeled “low”, *n* = 2805) comprised 71.2% of the children (see Fig. 1). Due to loss to follow-up at age 15, as sensitivity analysis, we reran the analyses specifying the trajectory groups including only individuals who had information about conduct problems at all points (*n* = 1760), using a four-group model (BIC-9024,06) and cubic term as the best fitting model. These analyses also showed high APP values (all APPs ranging from 0.80 to 0.94) and the four trajectories had a similar shape and prevalence rate when compared to the main model, despite there being smaller absolute numbers in each group (see Supplementary Fig. 1).

### Description of early risk factors according to conduct problem trajectories

All risk factors were significantly related to the four conduct problem trajectories, with the early-onset persistent trajectory generally being exposed to the highest levels of risk, followed by the adolescence-onset and then childhood-limited trajectories (see Table 2). This pattern was observed across the sociodemographic risk domain (low maternal age, low maternal schooling, low family income, high crowding, and living without a father figure), prenatal risks (maternal smoking and alcohol use), maternal depression, and parenting risk factors (maternal depression, low child stimulation, harsh parenting), exposure to trauma (interpersonal trauma), and child neurocognitive and mental health risk (low child development score, low IQ, and more attention problems).

**Table 2:** Exposure to early risk factors according to specific conduct problem trajectories

Early risk factors	Conduct problem trajectory				
	Low *n* (%)	Adolescence-onset *n* (%)	Childhood-limited *n* (%)	Early-onset persistent *n* (%)	*p-*value^a^
*Sociodemographic risk factors*					
*Maternal age (years)*					< 0.001
< 19	340 (12.1)	44 (15.4)	145 (20.8)	27 (18.0)	
≥ 19	2,462 (87.9)	242 (84.6)	552 (79.2)	123 (82.0)	
*Maternal schooling (years)*					< 0.001
0–4	238 (12.2)	80 (29.6)	127 (18.9)	42 (28.8)	
5–8	940 (35.0)	106 (39.3)	285 (42.5)	67 (45.9)	
≥ 9	1,415 (52.7)	84 (31.1)	259 (38.6)	37 (25.3)	
*Family income (in tertiles)*					< 0.001
1st (poorest)	838 (31.1)	129 (47.6)	286 (42.6)	85 (58.2)	
2nd	846 (31.4)	87 (32.1)	222 (33.1)	36 (24.7)	
3rd (richest)	1,013 (37.6)	55 (20.3)	163 (24.3)	25 (17.1)	
Household crowding (individuals per bedroom)					< 0.001
> 2	1,332 (49.4)	174 (64.2)	413 (61.6)	93 (63.7)	
≤ 2	1,364 (50.6)	97 (35.8)	258 (38.5)	53 (36.3)	
*Living without a father figure*					< 0.001
Yes	541 (20.1)	77 (28.4)	182 (27.1)	74 (50.7)	
No	2,156 (79.9)	194 (71.6)	489 (72.9)	72 (49.3)	
*Prenatal risk factors*					
Maternal smoking					< 0.001
Yes	633 (22.6)	115 (40.2)	251 (36.0)	69 (46.0)	
No	2,171 (77.4)	171 (59.8)	446 (64.0)	81 (54.0)	
*Maternal alcohol consumption*					< 0.001
Yes	79 (2.8)	17 (5.9)	21 (3.0)	12 (8.0)	
No	2,715 (97.2)	269 (94.1)	676 (97.0)	138 (92.0)	
*Maternal mental health*					
*Maternal depression*					< 0.001
Yes	349 (13.1)	82 (30.5)	185 (28.2)	54 (37.2)	
No	2,315 (86.9)	187 (69.5)	472 (71.8)	91 (62.8)	
*Parenting risk factors*					
*Child stimulation score*					< 0.001
0–2 (lower)	637 (23.7)	93 (34.6)	228 (33.9)	55 (37.9)	
3	791 (29.4)	92 (34.2)	198 (29.5)	47 (32.4)	
4–5 (higher)	1,261 (46.9)	84 (31.2)	246 (36.6)	43 (29.7)	
*Harsh parenting (in tertiles)*					< 0.001
3rd (worse)	588 (23.9)	109 (43.3)	303 (49.0)	76 (58.5)	
2nd	860 (35.0)	78 (31.0)	190 (30.7)	32 (24.6)	
1st (better)	1,013 (41.2)	65 (25.8)	125 (20.2)	22 (16.9)	
*Childhood trauma*					
*Interpersonal trauma*					< 0.001
Present	102 (4.0)	10 (3.9)	53 (8.1)	18 (13.3)	
Absent	2,439 (96.0)	245 (96.1)	599 (91.9)	117 (86.7)	
*Child neurocognitive risk factors*					
*Low child development*					< 0.001
Yes	252 (9.4)	33 (12.2)	111 (16.6)	26 (17.8)	
No	2,443 (90.7)	238 (87.8)	559 (83.4)	120 (82.2)	
*Low IQ*					< 0.001
Yes	619 (24.6)	118 (47.0)	254 (39.9)	70 (52.6)	
No	1,894 (75.4)	133 (53.0)	382 (60.1)	63 (47.4)	
*Child mental health*					
Attention problems subscale (score/ mean[sd])	1.96 (0.04)	3.61 (0.15)	4.13 (0.10)	5.94 (0.23)	< 0.001

2004 Pelotas (Brazil) Birth Cohort, *N* = 3938

*sd* standard deviation

^a^Pearson’s chi-squared test for any difference across trajectory groups

### Associations between early risk factors and conduct problem trajectories

Table 3 shows adjusted multinomial regression associations between early risk factors and elevated conduct problem trajectories (i.e., childhood-limited, adolescent-onset, and early-onset persistent group) using the “low” trajectory as a reference group. The early risk factors are organized in five hierarchical levels, with a separate model estimated for each level, adjusting for variables in preceding levels. All socioeconomic risk factors (see Table 3, level 1) significantly distinguished the four trajectory groups (*p* < 0.001 contrasting all trajectories), and (apart from maternal age) predicted increased risk for all three elevated conduct problem trajectories (childhood limited, adolescent limited and early-onset persistent) compared to the low trajectory.

**Table 3:** Adjusted associations between early risk factors, organized in five ecological levels, and three conduct problem trajectories (versus low problems) in multinomial logistic regression

Level	Early risk factors	Conduct problem trajectories						*p*-value test between all trajectories
		Childhood-limited *vs.* Low		Adolescent-onset *vs.* Low		Early-onset persistent *vs.* Low		
		OR_adj_ (95%CI)^a^	*p*-value	OR_adj_ (95%CI)^a^	*p*-value	OR_adj_ (95%CI)^a^	*p*-value	
1	*Sociodemographic risk factors*							
	*Maternal age (years)*							< 0.001
	< 19	1.64 (1.30; 2.05)	< 0.001	1.17 (0.81; 1.67)	0.402	1.21 (0.77; 1.90)	0.411	
	≥ 19	*Ref*		*Ref*		*Ref*		
	*Maternal schooling (years)*							< 0.001
	0–4	1.64 (1.26; 2.14)	< 0.001	3.08 (2.14; 4.44)	< 0.001	3.56 (2.14; 5.90)	< 0.001	
	5–8	1.30 (1.06; 1.59)	0.013	1.50 (1.09; 2.07)	0.013	2.09 (1.34; 3.25)	0.001	
	≥ 9	*Ref*		*Ref*		*Ref*		
	*Family income (in tertiles)*							0.002
	1st (poorest)	1.45 (1.14; 1.85)	0.003	1.61 (1.11; 2.34)	0.012	1.83 (1.11; 3.04)	0.018	
	2nd	1.28 (1.01; 1.62)	0.037	1.36 (0.94; 1.98)	0.100	1.12 (0.65; 1.93)	0.671	
	3rd (richest)	*Ref*		*Ref*		*Ref*		
	*Household crowding (individuals per bedroom)*							0.001
	> 2	1.37 (1.13; 1.65)	0.001	1.35 (1.02; 1.80)	0.035	1.41 (0.97; 2.06)	0.072	
	≤ 2	*Ref*		*Ref*		*Ref*		
	*Living without a father figure*							< 0.001
	Yes	1.45 (1.18; 1.77)	< 0.001	1.57 (1.17; 2.10)	0.003	4.00 (2.81; 5.69)	< 0.001	
	No	*Ref*		*Ref*		*Ref*		
2	*Prenatal risk factors*							
	*Maternal smoking*							< 0.001
	Yes	1.68 (1.38; 2.03)	< 0.001	1.73 (1.31; 2.28)	< 0.001	1.85 (1.29; 2.66)	0.001	
	No	*Ref*		*Ref*		*Ref*		
	*Maternal alcohol consumption*							0.058
	Yes	0.82 (0.49; 1.37)	0.452	1.74 (0.99; 3.06)	0.056	1.81 (0.90; 3.63)	0.095	
	No	*Ref*		*Ref*		*Ref*		
3	*Maternal mental health*							
	*Maternal depression*							< 0.001
	Yes	2.21 (1.79; 2.73)	< 0.001	2.19 (1.63; 2.94)	< 0.001	2.67 (1.84; 3.87)	< 0.001	
	No	*Ref*		*Ref*		*Ref*		
4	*Parenting risk factors*							
	*Child stimulation score*							0.070
	0–2 (lower)	1.33 (1.04; 1.69)	0.023	1.42 (0.99; 2.05)	0.059	1.53 (0.92; 2.53)	0.100	
	3	1.04 (0.82; 1.33)	0.737	1.38 (0.97; 1.97)	0.077	1.52 (0.93; 2.51)	0.097	
	4–5 (higher)	*Ref*		*Ref*		*Ref*		
	Harsh parenting (in tertiles)							< 0.001
	3rd (worse)	4.10 (3.19; 5.28)	< 0.001	3.09 (2.16; 4.41)	< 0.001	7.27 (4.22; 12.54)	< 0.001	
	2nd	1.83 (1.41; 2.38)	< 0.001	1.54 (1.06; 2.23)	0.022	2.17 (1.19; 3.97)	0.012	
	1st (better)	*Ref*		*Ref*		*Ref*		
	*Childhood trauma*							
	*Interpersonal trauma*							< 0.001
	Present	1.62 (1.08; 2.43)	0.019	0.89 (0.43; 1.82)	0.750	3.43 (1.89; 6.21)	< 0.001	
	Absent	*Ref*		*Ref*		*Ref*		
5	*Child neurocognitive risk factors*							
	*Low child development*							0.182
	Yes	1.34 (0.97; 1.86)	0.080	0.91 (0.58; 1.44)	0.693	1.49 (0.84; 2.66)	0.172	
	No	*Ref*		*Ref*		*Ref*		
	*Low IQ*							0.108
	Yes	1.20 (0.94; 1.54)	0.148	1.44 (1.04; 1.99)	0.028	1.32 (0.83; 2.10)	0.242	
	No	*Ref*		*Ref*		*Ref*		
	*Child mental health*							< 0.001
	*Attention problems subscale (score)*	1.50 (1.43; 1.58)	< 0.001	1.34 (1.25; 1.43)	< 0.001	1.86 (1.71; 2.03)	< 0.001	

2004 Pelotas (Brazil) Birth Cohort, *N* = 3938

*OR* Odds ratio; 95% CI 95% confidence interval

^a^Five models were estimated, one for each Level. Adjusted models include variables in the same Level and variables from preceding Levels where *p* < 0.20

Regarding prenatal risk factors (see Table 3, level 2), there was little evidence that exposure to alcohol consumption during pregnancy predicted conduct problem trajectory membership (*p* = 0.058, comparing between all trajectories). However, children exposed to prenatal maternal smoking had almost twice the risk for elevated conduct problems (ORs ranging from 1.68 to 1.85; *p *< 0.001 contrasting all trajectories). Similarly, children who were exposed to maternal depression (see Table 3, level 3) were more than twice as likely to show elevated conduct problems in childhood and/or adolescence (ORs ranging from 2.19 to 2.67; *p* < 0.001 contrasting all trajectories).

Regarding parenting risk factors (see Table 3, level 4), there was little evidence that child cognitive stimulation predicted conduct problem trajectory membership (*p* = 0.070, comparing between all trajectories). However, harsh parenting predicted increased risk for elevated conduct problem trajectories up to sevenfold (ORs ranging from 1.54 to 7.27; *p* < 0.001 contrasting all trajectories). Children who were exposed to interpersonal trauma were around three times more likely to have an early-onset persistent trajectory of conduct problems (OR = 3.43), followed by a smaller increase in risk for a childhood-limited trajectory (OR = 1.62) (*p* < 0.001, contrasting all trajectories)—however, exposure to interpersonal trauma was not associated with adolescent-onset conduct problems (*p* = 0.750 for specific contrast with the low trajectory).

Child neurocognitive risk factors did not significantly predict conduct problem trajectories overall (see Table 3, level 5), although low IQ did predict increased risk for adolescent-onset conduct problems specifically (versus the low trajectory, OR = 1.44, 95%CI = 1.04, 1.99). Finally, children with attention problems in early childhood were more likely to have elevated conduct problems trajectories (ORs ranging from 1.34 to 1.86; *p* < 0.001, contrasting all trajectories) (see Table 3 for full details).

Table 4 compares between specific conduct problem trajectories to see if early risk factors could distinguish a trajectory of early-onset persistent conduct problems from either childhood-limited or adolescence-onset conduct problems. Six risk factors significantly (*p* < 0.05) predicted early-onset persistent conduct problems versus the childhood-limited trajectory: low maternal education (OR = 2.17 for mothers with 0–4 years of schooling), absent father figure (OR = 2.77), and maternal drinking in pregnancy (OR = 2.20), harsh parenting (OR = 1.77, for top tercile), exposure to interpersonal trauma (OR = 2.11), and attention problems (OR = 1.24). Four risk factors significantly distinguished early-onset persistent conduct problems compared to the adolescent-onset trajectory: absent father figure (OR = 2.55), harsh parenting (OR = 2.36, for top tercile), exposure to interpersonal trauma (OR = 3.85), and attention problems (OR = 1.39) (see Table 4 for full details).

**Table 4:** Adjusted associations between early risk factors, organized in five ecological levels, and contrasts of specific conduct problem trajectory pairs in multinomial logistic regression

Level	Early risk factors	Conduct problem trajectories			
		Early-onset persistent vs. Childhood-limited		Early-onset persistent vs. Adolescent-onset	
		OR_adj_ (95%CI)^a^	*p*-value	OR_adj_ (95%CI)^a^	*p*-value
1	*Sociodemographic risk factors*				
	*Maternal age (years)*				
	< 19	0.74 (0.46; 1.19)	0.210	1.04 (0.60; 1.79)	0.894
	≥ 19	*Ref*		*Ref*	
	*Maternal schooling (years)*				
	0–4	2.17 (1.26; 3.72)	0.005	1.15 (0.64; 2.09)	0.638
	5–8	1.61 (1.01; 2.57)	0.047	1.39 (0.82; 2.36)	0.224
	≥ 9	*Ref*		*Ref*	
	*Family income (in tertiles)*				
	1st (poorest)	1.27 (0.74; 2.17)	0.389	1.14 (0.62; 2.08)	0.680
	2nd	0.88 (0.49; 1.55)	0.651	0.82 (0.44; 1.56)	0.554
	3rd (richest)	*Ref*		*Ref*	
	*Household crowding (individuals per bedroom)*				
	> 2	1.03 (0.69; 1.55)	0.869	1.04 (0.66; 1.64)	0.852
	≤ 2	*Ref*		*Ref*	
	*Living without a father figure*				
	Yes	2.77 (1.89; 4.04)	< 0.001	2.55 (1.66; 3.93)	< 0.001
	No	*Ref*		*Ref*	
2	*Prenatal risk factors*				
	*Maternal smoking*				
	Yes	1.10 (0.75; 1.62)	0.610	1.07 (0.70; 1.64)	0.758
	No	*Ref*		*Ref*	
	*Maternal alcohol consumption*				
	Yes	2.20 (1.01; 4.83)	0.048	1.04 (0.46; 2.35)	0.923
	No	*Ref*		*Ref*	
3	*Maternal mental health*				
	*Maternal depression*				
	Yes	1.21 (0.82; 1.79)	0.337	1.22 (0.79; 1.89)	0.373
	No	*Ref*		*Ref*	
4	*Parenting risk factors*				
	*Child stimulation score*				
	0–2 (lower)	1.15 (0.68; 1.94)	0.600	1.07 (0.60; 1.93)	0.812
	3	1.46 (0.87; 2.46)	0.152	1.11 (0.62; 1.98)	0.734
	4–5 (higher)	*Ref*		*Ref*	
	*Harsh parenting (in tertiles)*				
	3rd (worse)	1.77 (1.00; 3.14)	0.049	2.36 (1.27; 4.37)	0.007
	2nd	1.18 (0.63; 2.23)	0.600	1.41 (0.71; 2.79)	0.323
	1st (better)	*Ref*		*Ref*	
	*Childhood trauma*				
	*Interpersonal trauma*				
	Present	2.11 (1.14; 3.92)	0.017	3.85 (1.65; 8.97)	0.002
	Absent	*Ref*		*Ref*	
5	*Child neurocognitive risk factors*				
	*Low child development*				
	Yes	1.12 (0.63; 1.97)	0.707	1.64 (0.84; 3.18)	0.144
	No	*Ref*		*Ref*	
	*Low IQ*				
	Yes	1.10 (0.69; 1.75)	0.690	0.92 (0.54; 1.54)	0.743
	No	*Ref*		*Ref*	
	*Child mental health*				
	Attention problem subscale (score)	1.24 (1.14; 1.35)	< 0.001	1.39 (1.27; 1.53)	< 0.001

2004 Pelotas (Brazil) Birth Cohort, *N* = 3938

*OR* Odds ratio, 95% CI 95% confidence interval

^a^Five models were estimated, one for each Level. Adjusted models include variables in the same Level and variables from preceding Levels where *p* < 0.20

## Discussion

To our knowledge, this is the first study of conduct problem trajectories from early childhood into adolescence in the Global South. We identified four developmental trajectories of conduct problems in a large Brazilian birth cohort: one low-problem group, and three groups with elevated problems including a childhood-limited group, an early-onset persistent group, and an adolescence-onset group. A wide range of early psychosocial risk factors predicted all three types of elevated conduct problem trajectories, compared to low conduct problems. Importantly, some early child characteristics and environmental factors also predicted a higher risk for early-onset persistent conduct problems compared to both childhood-limited and adolescence-onset trajectories. Overall, the results are quite consistent with findings from studies in HICs, with children on the early-onset persistent trajectory being exposed to the highest levels of risk.

Prior evidence has shown that a pattern of early-onset persistent conduct problems is strongly associated with a wide variety of negative outcomes in later life [7]. Our results, based on a Brazilian population-based sample, support previous findings on the importance of a range of early environmental factors and child characteristics for this early-onset persistent pattern of conduct problems [47]. Thus, compared to children with low conduct problems, children with an early-onset persistent trajectory were more likely to have been exposed to low maternal schooling, low family income, living without a father figure, maternal smoking in pregnancy, maternal and child mental health problems, harsh parenting, and interpersonal trauma. Several of these risk factors also helped distinguish this pattern of persistent conduct problems from childhood-limited or adolescent-onset trajectories. Most important in this regard were exposure to interpersonal trauma, living without a father, and child attention difficulties—which were particularly elevated for the early-onset group, compared to all others. Possible mechanisms linking trauma and persistent behavior problems include long-lasting biological alterations related to a threat, and psychological changes in attachment relations and social information-processing, leading to impulsive or dissociative coping styles or cognitive patterns encouraging later aggression [48]. Also, child maltreatment may lead to ruptures in the family environment (e.g., being placed in foster care). Living without a father figure may represent several different types of risk for children, including prior conflict in parental relationships, as well as reduced social support, experiences of abandonment, and lack of a positive male role model for prosocial development [49].

The pathway linking attention problems with persistently elevated conduct problems may be explained by children struggling to develop in learning environments that promote prosocial behavior, and in interaction difficulties within family and social relationships [47, 50]. According to Moffitt’s theory, the effects of neuropsychological difficulties are likely to be observed in difficult early carer–child interactions and accumulate across other social settings with teachers and peers to produce problem behaviors that persist into adolescence and beyond, knifing off prosocial opportunities [7].

Most early risk factors in the current study also predicted adolescent-onset patterns of conduct problems (compared to low conduct problems), albeit with generally weaker associations than for the early-onset persistent group. The relevance of early family environment (e.g., maternal schooling, family income, living without a father figure, prenatal smoking and alcohol use, maternal depression, and harsh parenting), and child neurodevelopment (low IQ and attention difficulties) for adolescent-onset conduct problems questions the qualitative nature of Moffitt’s developmental taxonomy theory. This theory and findings from some prior studies, most notably the Dunedin study, suggest that life-course persistent and adolescent-onset conduct problems have entirely different etiologies [13, 18]. Fairchild et al. [51] suggested that the developmental taxonomic theory requires revision, because differences between these trajectories may be quantitative (in the number and magnitude of risk factors), rather than qualitative (complete specificity of risk factors for each type of trajectory). The quantitative differences in our study support this revision, as well as a recent systematic review of prospective longitudinal studies [15] finding that, while a life-course persistent trajectory is characterized by greater overall exposure to risk across family and individual domains, such influences also influence adolescent-onset conduct problems. These findings do not negate the possibility of adolescent-specific peer influences and the “maturity gap”, as proposed by Moffitt’s theory, but suggest that they are not the only relevant factors for children first manifesting conduct problems in adolescence.

Regarding the childhood-limited trajectory, all early risk factors were significant predictors of this course of conduct problems, except maternal alcohol consumption in pregnancy and poor child development. However, again associations were weaker than for the early-onset persistent trajectory—similar to the findings from the ALSPAC cohort in the UK [52]. Consistent with the lack of an association between maternal alcohol consumption and childhood-limited conduct problems in the current study, Mendelian randomization analyses in ALSPAC showed that alcohol use contributed to increased risk for early‐onset persistent conduct problems, with potential causal effects, but not childhood-limited problems [53].

Harsh parenting had the largest association with childhood-limited conduct problems in the current study. However, this association is particularly difficult to interpret given the likelihood of bidirectional effects, from child behavior problems to harsh parenting, as well as vice versa [50, 54]. Interestingly, there was little evidence of differences in risk factor exposure between children with low conduct problems and those with a childhood-limited trajectory in terms of early child development or IQ. This may mean that, although challenging behavior in early childhood is provoked by difficult early home environments, normative cognitive development, and lower levels of trauma exposure, helped children learn out of such difficulties as they moved from childhood into adolescence (unlike children following an early-onset persistent path).

Our study addressed an important gap in the literature by identifying developmental trajectories of conduct problems in Brazil, a middle-income country, with higher levels of economic disadvantage, inequality and violence, than in many high-income countries where most prior research has been conducted. This revealed similar longitudinal patterns of conduct problems, in four distinct groups, as previously identified in studies elsewhere, and then documented similar types of bio-psycho-social risk factors influencing those trajectories. This is significant in terms of strengthening the evidence base on a range of early bio-psycho-social influences on behavioral development, pointing to robust findings across very different social environments. However, there were differences in the size of the trajectory groups estimated in this Brazilian study compared to previous studies—smaller proportions of this sample had early-onset persistent or childhood-limited conduct problem trajectories than elsewhere. This difference should be considered cautiously considering methodological as well as contextual differences across studies. In the current study, we applied widely used instruments (the CBCL and SDQ) to measure conduct problems and included both boys and girls from a whole population; some other samples are defined as high risk within a population. The trajectory analyses were based on conduct problems z-scores, to allow comparisons over time using two instruments in our sample, making direct comparisons of estimates difficult with other studies that used different methods. Methodological differences or social context may explain the larger proportion of children in this study classified in the low conduct problem group (71.2%) compared to several prior studies in HICs of children of a similar age (3–17 years; rates ranging between 48.0% and 64.3%) [52, 55, 56]. With more “low conduct children” in the current study, there were necessarily fewer children classified in elevated groups, and fewer children had an early-onset persistent pattern of conduct problems (3.8% of children), compared to several studies in HICs [52, 55–60]. Also, the adolescence-onset group was smaller in our study (7.3%) compared to several others reporting rates ranging from 11.8% [52] to 15.0% [55, 58]. However, the size of the childhood-limited group (17.7%) is similar to several others: 12.0% in a Belgian population-based sample [55], 15.9% in one UK study [58], and 23.2% in a second UK study [56].

There are several methodological strengths of the current study, including the population-based, prospective design, and the use of internationally validated measures of mental health. However, the following limitations should be considered when interpreting the findings. First, there was high attrition at the 15-year follow-up due to the Covid-19 pandemic. This problem is somewhat overcome because there were few differences between those included and those who were not assessed at age 15, suggesting that any attrition bias is likely to be small, however, the attrition is not completely random and unmeasured differences may also be apparent. Using a censored normal model, 95% of participants had at least 3 times points of data, but about half were missing age 15 data given the pandemic. Furthermore, this method—full information maximum likelihood—has been shown to produce unbiased parameter estimates compared to other methods [61]. The trajectory model including individuals with information on all points was tested in sensitivity analysis, and no differences were found regarding the number and shape of trajectories reinforcing the idea that there is limited bias due to the losses at the age 15 follow-up. A second limitation was that in order to use data on conduct problems at age 4 years (when only the CBCL had been applied) as well as ages 6, 11, and 15 (when the SDQ was applied), we constructed conduct problem trajectories from 4 to 15 based on data from two instruments. Although many items in these two instruments are similar, and the CBCL externalizing subscale is strongly correlated with SDQ conduct problems [33], ideally the same instrument would have been used throughout the study. Third, we relied on caregiver reports, which may have underestimated the level of conduct problems, particularly at later time points. A multi-informant approach, including teacher and/or self-report, may have captured symptoms not recognized by caregivers. Finally, these are all, of course, observational associations, and causal processes cannot be inferred from the results.

In conclusion, this Brazilian study found similar patterns of conduct problem trajectories (number and shape), and similar key risk factors as in many prior studies elsewhere, despite considerable cultural and social differences. Our findings contribute to a growing evidence base on how early risk factors can affect the development of conduct problems, highlighting both the contribution of early risk factors for all trajectories identified, as well as the particular importance of exposures such as interpersonal trauma for more persistent early-onset conduct problems.

## Supplementary Information

Below is the link to the electronic supplementary material.

Supplementary file1 (DOCX 55 KB)

## Data Availability

The data and all analytical scripts supporting the findings of this study are available on request from the first author (TM-S).
