# Supplementary material for: Early risk factors for conduct problem trajectories from childhood to adolescence: the 2004 Pelotas (BRAZIL) Birth Cohort
Source: Eur Child Adolesc Psychiatry. 2023 Apr 25;33(3):881–95. doi: 10.1007/s00787-023-02178-9 (PMC10126565; doi:10.1007/s00787-023-02178-9)
Supplement: Supplementary file 1 — Supplementary file1 (DOCX 55 KB) [file 787_2023_2178_MOESM1_ESM.docx]

**Supplementary Material**

**EARLY RISK FACTORS FOR CONDUCT PROBLEM TRAJECTORIES FROM CHILDHOOD TO ADOLESCENCE: THE 2004 PELOTAS (BRAZIL) BIRTH COHORT**

Thais Martins-Silva ^1,2^, Andreas Bauer ^1,2^, Alicia Matijasevich ^2,3^, Tiago N. Munhoz ^2^, Aluísio J. D. Barros ^2,4^, Iná S. Santos ^2,5^, Luciana Tovo-Rodrigues ^1,2^, Joseph Murray ^1,2*^

*^1^Human Development and Violence Research Centre (DOVE), Federal University of Pelotas, Pelotas, Brazil*

*^2^Post-Graduate Program in Epidemiology, Federal University of Pelotas, Pelotas, Brazil*

*^3^Departamento de Medicina Preventiva, Faculdade de Medicina FMUSP, Universidade de São Paulo, Brasil*

*^4^International Center for Equity in Health, Federal University of Pelotas, Pelotas, Brazil*

*^5^Postgraduate Program in Pediatrics and Child Health, School of Medicine, Pontifical Catholic University of Rio Grande do Sul, Porto Alegre, Brazil*

*Correspondence to: Joseph Murray, Human Development and Violence Research Centre (DOVE), Federal University of Pelotas, Pelotas, Brazil, [j.murray@doveresearch.org](mailto:j.murray@doveresearch.org)

**Supplementary Table 1.** Summary of early risk factors for specific conduct problem trajectories included in the analysis.

| **Level** | **Early risk factors group** | **Measure** | **Follow-up** | **Coded as** | **N of included sample** |
| --- | --- | --- | --- | --- | --- |
| 1 | Sociodemographic risk factors | Maternal age | Perinatal | <19 or ≥19 years | 3,935 |
|  |  | Maternal schooling | 4-years | 0-4, 5-8 or ≥ 9 years | 3,770 |
|  |  | Family income | 4-years | In tertiles | 3,785 |
|  |  | Household crowding | 4-years | ≤2 or >2 individuals per bedroom | 3,784 |
|  |  | Living without a father figure | 4-years | No, or yes | 3,785 |
| 2 | Prenatal risk factors | Maternal smoking | Perinatal | No, or yes | 3,937 |
|  |  | Maternal alcohol consumption | Perinatal | No, or yes | 3,937 |
| 3 | Maternal mental health | Maternal depression | 4-years | No or yes; Cutoff point of ≥13 | 3,735 |
| 4 | Parenting risk factors | Child stimulation score | 2-years | 0-2; 3 or 4-5 points | 3,775 |
|  |  | Harsh parenting | 6-years | In tertiles | 3,461 |
|  | Childhood trauma | Interpersonal trauma | 6-years | Present or absent; At least one affirmative answer | 3,583 |
| 5 | Child neurocognitive risk factors | Low child development | 4-years | No, or yes; belonging to the first decile | 3,782 |
|  |  | Low IQ | 6-years | Yes or no, the cutoff point of <70 | 3,533 |
|  | Child mental health | Attention problems subscale | 4-years | Continuous | 3,749 |

IQ: intelligence quotient

| **Supplementary Table 2.** Description of potential early risk factors for conduct problem trajectories contrasts between those participants included and not included in the analysis. 2004 Pelotas (Brazil) Birth Cohort | | | | | |
| --- | --- | --- | --- | --- | --- |
| **Variables** | **Included** (*n*=3,938) | | **Not included** (*n*=293) | | ***p*-value**^a^ |
|  | ***n*** | **% (95% CI) *or* mean (sd)** | ***n*** | **% (95% CI) *or*  mean (sd)** |  |
| Sex |  |  |  |  | 0.903 |
| Male | 2,044 | 51.9 (50.3; 53.5) | 151 | 51.5 (45.8; 57.2) |  |
| Female | 1,894 | 48.1 (46.5; 49.7) | 142 | 48.5 (42.8; 54.2) |  |
| ***Sociodemographic risk factors*** |  |  |  |  |  |
| Maternal age (years) |  |  |  |  | 0.392 |
| <19 | 556 | 14.1 (13.1;15.3) | 36 | 12.3 (9.0;16.6) |  |
| ≥19 | 3.379 | 85.9 (84.7;86.9) | 256 | 87.7 (83.4;91.0) |  |
| Maternal schooling (years) |  |  |  |  | 0.054 |
| 0 – 4 | 577 | 15.3 (14.2;16.5) | 4 | 30.8 (12.0;59.1) |  |
| 5 – 8 | 1,398 | 37.1 (35.6;38.6) | 7 | 53.9 (28.2;77.6) |  |
| ≥9 | 1,795 | 47.6 (46.0;49.2) | 2 | 15.4 (3.9;45.1) |  |
| Family income (in tertiles) |  |  |  |  | 0.968 |
| 1^st^ (poorest) | 1,338 | 35.4 (33.8;36.9) | 5 | 35.7 (15.7;62.4) |  |
| 2^nd^ | 1,191 | 31.5 (30.0;33.0) | 4 | 28.6 (11.1;56.1) |  |
| 3^rd^ (richest) | 1,256 | 33.2 (31.7;34.7) | 5 | 35.7 (15.7;62.4) |  |
| Household crowding (individuals per bedroom) |  |  |  |  | 0.247 |
| ≤2 | 1,772 | 46.8 (45.2;48.4) | 4 | 30.8 (12.0;59.1) |  |
| >2 | 2,012 | 53.2 (51.6;54.8) | 9 | 69.2 (40.9;88.0) |  |
| Living without a father figure |  |  |  |  | 0.883 |
| No | 2.911 | 76.9 (75.5;78.2) | 11 | 78.6 (50.6;92.9) |  |
| Yes | 874 | 23.1 (21.8;24.5) | 3 | 21.4 (7.1;49.4) |  |
| ***Prenatal risk factors*** |  |  |  |  |  |
| Maternal smoking |  |  |  |  | 0.061 |
| No | 2,869 | 72.9 (71.5;74.2) | 198 | 67.8 (62.2;72.9) |  |
| Yes | 1,068 | 27.1 (25.8;28.5) | 94 | 32.2 (27.1;37.8) |  |
| Maternal alcohol consumption |  |  |  |  | 0.651 |
| No | 3.808 | 96.7 (96.1;97.2) | 281 | 96.2 (93.3;97.9) |  |
| Yes | 129 | 3.3 (2.8;3.9) | 11 | 3.8 (2.1;6.7) |  |
| ***Maternal mental health*** |  |  |  |  |  |
| Maternal depression |  |  |  |  | 0.811 |
| No | 3,065 | 82.1 (81.0;83.3) | 11 | 84.6 (54.9;96.1) |  |
| Yes | 670 | 17.9 (16.7;19.2) | 2 | 15.4 (3.9;45.1) |  |
| ***Parenting risk factors*** |  |  |  |  |  |
| Child stimulation score |  |  |  |  | 0.872 |
| 0 – 2 (lower) | 1,013 | 26.8 (25.4;28.3) | 24 | 28.6 (19.9;39.1) |  |
| 3 | 1,128 | 29.9 (28.4;31.4) | 26 | 31.0 (22.0;41.6) |  |
| 4 – 5 (higher) | 1,634 | 43.3 (41.7;44.9) | 34 | 40.5 (30.5;51.3) |  |
| Harsh parenting (in tertiles) |  |  |  |  | - |
| 1^st^ (better) | 1,225 | 35.4 (33.8;37.0) | 0 | - |  |
| 2^nd^ | 1,160 | 33.5 (32.0;35.1) | 0 | - |  |
| 3^rd^ (worse) | 1,076 | 31.1 (30.0;32.7) | 0 | - |  |
| ***Childhood trauma*** |  |  |  |  |  |
| Interpersonal trauma |  |  |  |  | - |
| Absent | 3,400 | 94.9 (94.1;95.6) | 0 | - |  |
| Present | 183 | 5.1 (4.4;5.9) | 0 | - |  |
| ***Child neurocognitive risk factors*** |  |  |  |  |  |
| Low child development |  |  |  |  | 0.711 |
| No | 3,360 | 88.8 (87.8;89.8) | 12 | 85.7 (57.3;96.4) |  |
| Yes | 422 | 11.2 (10.2;12.2) | 2 | 14.3 (3.6;42.7) |  |
| Low IQ |  |  |  |  | - |
| No | 2,472 | 70.0 (68.4; 71.4) | 1 | - |  |
| Yes | 1,061 | 30.0 (28.5; 31.6) | 0 | - |  |
| ***Child mental health*** |  |  |  |  |  |
| Attention problems subscale (score/ mean[sd]) | 3,749 | 2.62 (0.04) | 0 | - | - |
| 95% CI: 95% confidence interval; sd: standard deviation.  ^a^ Person’s chi-square test; | | | | | |

| **Supplementary Table 3.** Model fit statistics for conduct problem trajectories. 2004 Pelotas (Brazil) Birth Cohort. N=3,938 | | | | | | |
| --- | --- | --- | --- | --- | --- | --- |
| **Conduct problem trajectories** | **n (%)** | **APP (sd)** | **Parameter** | **β** | **SE** | ***p*-value** |
| Early-onset persistent | 150 (3.8) | 0.877 (0.168) | Intercept | -2.923 | 0.812 | *<0.001* |
|  |  |  | Linear | 1.836 | 0.311 | *<0.001* |
|  |  |  | Quadratic | -0.188 | 0.036 | *<0.001* |
|  |  |  | Cubic | 0.006 | 0.001 | *<0.001* |
| Adolescence-onset | 286 (7.3) | 0.828 (0.181) | Intercept | 7.720 | 0.631 | *<0.001* |
|  |  |  | Linear | -3.068 | 0.243 | *<0.001* |
|  |  |  | Quadratic | 0.379 | 0.028 | *<0.001* |
|  |  |  | Cubic | -0.013 | 0.001 | *<0.001* |
| Childhood-limited | 697 (17.7) | 0.776 (0.169) | Intercept | -2.392 | 0.553 | *<0.001* |
|  |  |  | Linear | 1.356 | 0.216 | *<0.001* |
|  |  |  | Quadratic | -0.167 | 0.024 | *<0.001* |
|  |  |  | Cubic | 0.006 | 0.001 | *<0.001* |
| Low | 2.805 (71.2) | 0.930 (0.121) | Intercept | 0.012 | 0.166 | 0.941 |
|  |  |  | Linear | -0.152 | 0.063 | *0.015* |
|  |  |  | Quadratic | 0.016 | 0.007 | *0.021* |
|  |  |  | Cubic | -0.001 | 0.0002 | *0.033* |

sd: standard deviation; se: standard error; β: Beta coefficient; APP: average posterior probability.

**Note:** Lines represent estimated (latent) change over time. Dots represent observed group means at each age (markers). The square, circle, diamond, and triangle represent the exact z-score of conduct problems in each trajectory at ages 4, 6, 11, and 15 years.

**Supplementary Figure 1**. Conduct problem trajectories from ages 4 to 15 years for children with valid outcome data at all four-time points (N=1,760) in the 2004 Pelotas (Brazil) Birth Cohort.
